# Visual Outcomes of Ultrathin-Descemet Stripping Endothelial Keratoplasty versus Descemet Stripping Endothelial Keratoplasty

**DOI:** 10.1155/2018/5924058

**Published:** 2018-11-01

**Authors:** Konstantinos Droutsas, Myrsini Petrelli, Dimitrios Miltsakakis, Konstantinos Andreanos, Anastasia Karagianni, Apostolos Lazaridis, Chrysanthi Koutsandrea, George Kymionis

**Affiliations:** ^1^First Department of Ophthalmology, National and Kapodistrian University of Athens, General Hospital “G. Gennimatas”, Athens, Greece; ^2^Department of Ophthalmology, Philipps University, Marburg, Germany; ^3^State Ophthalmology Clinic, General Hospital “G. Gennimatas”, Athens, Greece; ^4^Jules Gonin Eye Hospital, Faculty of Biology and Medicine, University of Lausanne, Lausanne, Switzerland

## Abstract

**Purpose:**

To examine the impact of graft thickness (GT) on postoperative visual acuity and endothelial cell density after ultrathin-Descemet stripping automated endothelial keratoplasty (UT-DSAEK) versus conventional DSAEK.

**Methods:**

The medical records of all patients who underwent DSAEK at our institute during a 2-year period were reviewed. After excluding subjects with low visual potential, 34 eyes were divided into two groups based on the postoperative GT as measured with anterior segment optical coherence tomography (AS-OCT): an UT-DSAEK group (GT ≤ 100 *μ*m, *n*=13 eyes) and a DSAEK group (GT > 100 *μ*m, *n*=21 eyes). The groups were compared with regard to best-corrected visual acuity (BCVA), subjective refraction, central corneal thickness (CCT), GT, and endothelial cell density (ECD).

**Results:**

Preoperative BCVA (logMAR) was 1.035 ± 0.514 and 0.772 ± 0.428 for UT-DSAEK and DSAEK, respectively (*P*=0.072). At 6 months postoperatively, BCVA was 0.088 ± 0.150 following UT-DSAEK and 0.285 ± 0.158 following DSAEK (*P*=0.001).

**Conclusion:**

DSAEK grafts with a thickness under 100 *μ*m offered better visual outcomes during the early postoperative period.

## 1. Introduction

Over the past decade, Descemet stripping automated endothelial keratoplasty (DSAEK) has surpassed penetrating keratoplasty (PK) as the preferred treatment method for patients with corneal endothelial dysfunction [1]. The numerous advantages of DSAEK over PK include the avoidance of an open sky procedure, absence of suture-related complications, better tectonic and refractive stability, and faster visual rehabilitation [2, 3].

In contrast to PK, where all layers of the host cornea are replaced, DSAEK represents an additive procedure, where a graft consisting of a layer of posterior donor stroma of variable thickness and a layer of healthy corneal endothelium is placed on the posterior surface of the host cornea. This increased corneal thickness may limit the visual outcome after DSAEK [4]. Thus, a trend has emerged [5–7] favouring thinner grafts (i.e., thin and UT-DSAEK) and even alternative surgical procedures, such as Descemet membrane endothelial keratoplasty (DMEK) and pre-Descemet's endothelial keratoplasty (PDEK).

To date, the evidence for differences in visual outcomes depending on GT remains controversial. A number of studies have demonstrated a positive correlation between GT and postoperative visual acuity after DSAEK [8–10], whereas others have not provided supporting data for this hypothesis [11–13]. Thus, the aim of this study was to further elucidate the possible impact of GT on postoperative visual acuity by comparing visual outcomes of UT-DSAEK to those of DSAEK.

## 2. Materials and Methods

The medical records of all patients who had undergone DSAEK surgery between October 2015 and August 2017 at a tertiary referral centre (General Hospital “G. Gennimatas,” Athens, Greece) were reviewed. Cases with low visual potential (e.g., glaucoma, retinal macular disease, corneal scars, amblyopia) or concurrent ocular surgery (e.g., phacoemulsification) were excluded. Failed or complicated procedures requiring reinterventions such as rebubbling or repeat DSAEK were also excluded. In bilateral cases, only the right was included in the study. All procedures were performed by 3 surgeons (K.D., D.M., and G.K.) with the same surgical technique [14] using precut grafts acquired from an eye bank network that uses a single-pass donor DSAEK graft preparation technique (https://www.sightlife.org/Resources/prepared-corneal-tissue). Notably, the surgeons did not request grafts of a specific thickness range; therefore, the grafts used were of random thickness.

All patients underwent laser iridotomy prior to surgery. The DSAEK procedure was performed under subtenon's block and involved placement of a 4.5 mm limbal incision (temporally in left eyes and nasally in right eyes), followed by three side ports at 1.00, 6.00, and 9.00. After filling the anterior chamber with air, stripping of the recipient's Descemet membrane was performed using a reverse Sinskey hook. Following removal of the anterior cap of the precut donor tissue, the posterior lamella was mounted on a silicon bank (Geuder AG, Heidelberg, Germany) with the endothelium facing up. The donor lenticule was trephined with an 8.0 to 8.5 mm punch (Katena, U.S.A.), based on the corneal diameter of the recipient. The graft was then loaded onto a Busin glide (Moria SA, Germany), and a fine intraocular forceps (Moria SA) was used to pull the graft through the limbal incision. Incision wounds were sutured using 10-0 nylon. Following this, the anterior was filled completely with 100% air. After 60 to 120 minutes, the patient was examined at the slit lamp and some air was released by gently pressing on a side port in the case of pupillary block.

Preoperative and postoperative best-corrected visual acuity (BCVA), subjective refraction, central corneal thickness (CCT), GT, and endothelial cell density (ECD) were recorded.

**Figure 1:**
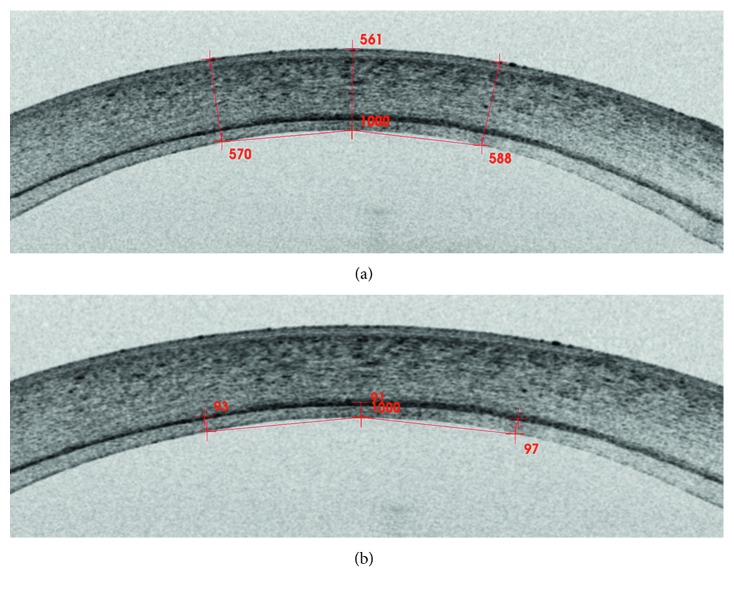
Thickness of a DSAEK graft as measured on an AS-OCT image. Both total (a) and graft (b) corneal thickness were measured at 5 points (at the point of intersection of the measurement's reference axis and the graft and at 1 mm distance in the superior, inferior, nasal, and temporal meridian). Here are depicted three horizontal points: at the point of intersection of the measurement's reference axis and the graft, and at 1 mm distance on both sides nasally and temporally to the centre.

CCT and GT were measured using swept-source anterior segment optical coherence tomography (AS-OCT) (DRI-OCT Triton, Topcon, Japan). More specifically, the average thickness of 5 points was recorded—measured at the vertex and at 1 mm located superiorly, inferiorly, temporally, and nasally to the vertex (Figure 1). In addition, four peripheral graft thickness measurements were taken, at two perpendicular axes within 3 mm from the centre, and the mean value of the latter measurements was calculated (*P*). Following this, the ratio of central to peripheral graft thickness (C : P) was calculated. Patients were divided into two groups based on postoperative GT: UT-DSAEK (GT ≤ 100 *μ*m) and DSAEK (GT > 100 *μ*m). Donor ECD was extracted from the donor information form, provided by the supplying eye bank. Postoperative ECD was measured with a noncontact specular microscope (EM-3000, Tomey, USA). BCVA was measured in Snellen and converted to a logarithm of the minimum angle of resolution (logMAR) in order to facilitate statistical analysis.

All data were collected with Excel software (version 14, Microsoft Corp.), analyzed with SPSS software (version 17.0, SPSS, Inc.,) and reported as central tendency and dispersion. Differences of means between the groups were assessed by the Mann–Whitney *U* test for independent samples. A *P* value less than 0.05 was considered statistically significant.

## 3. Results

The study enrolled 43 eyes of 43 patients. Thirteen eyes (*n*=13) fell within the UT-DSAEK group and 30 eyes (*n*=30) within the DSAEK group (Table 1). Postoperative GT was 87 ± 13 *μ*m and 145 ± 21 *μ*m following UT-DSAEK and DSAEK, respectively (*P* < 0.001). No significant difference between groups with respect to age and preoperative BCVA was observed (*P*≥0.072, Mann–Whitney *U* test). The mean corneal donor lenticule C:P ratio in the UT-DSAEK group was 0.65 ± 0.12 (*n*=12) and 0.86 ± 0.17 in the DSAEK group (*n*=6) (*P*=0.027). In the ultrathin group, mean logMAR improved from 1.035 (range 0.521 to 1.549) at baseline to 0.088 (range −0.062 to 0.238) at 6 months after surgery. In the DSAEK group, mean logMAR improved from 0.772 (range 0.344 to 1.2) at baseline to 0.285 (range −0.127 to 0.443) at 6 months. Postoperative logMAR was significantly better following UT-DSAEK (*P*=0.001).

A statistically significant moderate positive relationship between postoperative GT and logMAR values (*r*^2^=0.423, *P*=0.006, linear regression analysis) was found in the total group.

Donor ECD was significantly higher in the UT-DSAEK group (*P*=0.029). Postoperative ECD was 1403 ± 473 cells/mm^2^ and 1407 ± 411 cells/mm^2^ (*P*=0.715).

## 4. Discussion

DSAEK has replaced PK as the treatment of choice for corneal endothelial dysfunction. Thus, considering the increasing popularity of DSAEK, elucidating the contribution of various factors that influence the final visual outcome is highly significant. A number of factors, including donor-recipient interface, tissue irregularities, anterior corneal scarring, and high-order aberrations, have been suggested as influencing the visual outcome following DSAEK [15–17]. Moreover, a more regular posterior corneal surface has been shown to be achieved with thinner grafts, which, in turn, results in fewer, high-order aberrations. This reduction could explain the faster and better visual recovery observed with thinner DSAEK grafts [10, 18].

The aim of this retrospective study was to examine if GT less than 100 *μ*m is associated with better postoperative visual acuity, as this could provide guidance for optimizing DSAEK graft thickness. Therefore, we assessed the impact of GT on BCVA following UT-DSAEK versus conventional DSAEK and found significantly better BCVA after UT-DSAEK at 6 months after surgery, as well as a moderate positive correlation of GT with BCVA.

Our results are consistent with the findings of previous studies. Neff et al. conducted a retrospective study (*n*=33) and concluded that grafts that postoperatively were 131 *μ*m or thinner had a higher percentage of 20/25 and 20/20 final visual acuity results compared to grafts with a postoperative central GT greater than 131 *μ*m [9]. Pogorelov et al. correlated postoperative GT and BCVA 6 months after DSAEK and found a statistically significant relationship (*n*=15) [8]. Dickman et al. confirmed these results in a larger cohort of eyes without significant comorbidity (*n*=79) [10]. Acar et al. reported that thinner DSAEK grafts (GT < 150 *μ*m) are associated with better visual rehabilitation and less endothelial loss (*n*=37) [19]. The findings of a recent randomized multicenter clinical trial indicated that UT-DSAEK, compared to DSAEK, promotes faster and better visual acuity results with a similar endothelial cell loss at 1 year postoperatively (*n*=66) [20]. Finally, results from a relatively recent review of the literature and meta-analysis suggest a weak relationship between GT and BCVA following DSAEK [21].

Nevertheless, several other studies have not provided evidence supporting a clear association between GT and visual acuity following DSAEK [11–13, 22–24].

To our knowledge, there is currently no consensus on the basis upon which the categorization of GT should be done. The exact thickness defining UT, as opposed to conventional DSAEK, is not uniform in the literature and has been described variously as sub-130 *μ*m or sub-100 *μ*m [25, 26]. Moreover, some studies refer to the GT measurement immediately after graft preparation [13, 23, 27], while others use the measurement after surgery *in vivo* [10, 22, 24]. Here, we have used the definition of UT-DSAEK based on postoperative GT measurement as previously reported by others [4].

In order to assess donor corneal lenticule morphology, we calculated the C:P ratio of the DSAEK graft, an index that represents the ratio of the central graft thickness to the peripheral as formerly described [28]. Interestingly, the C:P ratio was found to be higher in the DSAEK group.

Different endothelial keratoplasty techniques have not shown significantly different ECL up to date. The slightly higher but not significant ECL in the UT-DSAEK group (47% versus 44% following UT-DSAEK and DSAEK, respectively) agrees with previous observations [20].

Limitations of this study include its retrospective nature and the small number of the studied eyes. Randomized, prospective studies with larger sample size are required in order to examine the relationship between both preoperative and postoperative donor thickness and postoperative vision and to confirm the theory that better visual outcomes can be achieved with the use of ultrathin DSAEK grafts.

## Figures and Tables

**Table 1:** Baseline and 6-month postoperative characteristics.

	Ultrathin DSAEK (GT ≤ 100 *μ*m)		DSAEK (GT > 100 *μ*m)		MWU test
	Mean ± SD	*n*	Mean ± SD	*n*	*P* value
Age (years)	72.7 ± 8.8	13	71.4 ± 7.3	30	0.701
Follow-up (months)	9.3 ± 4.6	13	7.4 ± 4.4	30	0.274
logMARpre	1.035 ± 0.514	13	0.772 ± 0.428	30	0.072
logMARpost	0.088 ± 0.150	13	0.285 ± 0.158	27	0.001^*∗*^
SEpost (D)	0.11 ± 0.54	13	0.31 ± 1.24	26	0.227
REFcylpost (D)	1.173 ± 0.874	13	1.154 ± 0.863	26	0.641
CCTpost (*μ*m)	579 ± 45	12	621 ± 56	16	0.039^*∗*^
Gtpost (*μ*m)	87 ± 13	13	145 ± 21	30	0.000^*∗*^
ECDpre (cells/mm^2^)	2698 ± 401	13	2457 ± 249	30	0.029^*∗*^
ECDpost (cells/mm^2^)	1403 ± 473	13	1407 ± 411	19	0.715
ECL (%)	47 ± 19	9	44 ± 16	19	0.418

^*∗*^Statistically significant; CCT: central corneal thickness; D: diopters; ECD: endothelial cell density; GT: graft thickness; logMAR: decadic logarithm of the minimal angle of resolution; mo: months; MWU: Mann–Whitney *U* test; post: postoperatively; pre: preoperatively; REFcyl: refractive cylinder; SE: spherical equivalent.

## Data Availability

The data used to support the findings of this study are available from the corresponding author upon request.
