# Erratum to “Visual Outcomes of Ultrathin-Descemet Stripping Endothelial Keratoplasty versus Descemet Stripping Endothelial Keratoplasty”

**DOI:** 10.1155/2019/4786431

**Published:** 2019-01-10

**Authors:** Konstantinos Droutsas, Myrsini Petrelli, Dimitrios Miltsakakis, Konstantinos Andreanos, Anastasia Karagianni, Apostolos Lazaridis, Chrysanthi Koutsandrea, George Kymionis

**Affiliations:** ^1^First Department of Ophthalmology, National and Kapodistrian University of Athens, General Hospital “G. Gennimatas”, Athens, Greece; ^2^Department of Ophthalmology, Philipps University, Marburg, Germany; ^3^State Ophthalmology Clinic, General Hospital “G. Gennimatas”, Athens, Greece; ^4^Jules Gonin Eye Hospital, Faculty of Biology and Medicine, University of Lausanne, Lausanne, Switzerland

In the article titled “Visual Outcomes of Ultrathin-Descemet Stripping Endothelial Keratoplasty versus Descemet Stripping Endothelial Keratoplasty” [1], the Authors' Contributions section was missing and should be added as follows:

## References

[1] Droutsas K., Petrelli M., Miltsakakis D. (2018). Visual outcomes of ultrathin-descemet stripping endothelial keratoplasty versus descemet stripping endothelial keratoplasty. *Journal of Ophthalmology*.

